# The effect of food insecurity during college on graduation and type of degree attained: evidence from a nationally representative longitudinal survey

**DOI:** 10.1017/S1368980021003104

**Published:** 2022-02

**Authors:** Julia A Wolfson, Noura Insolera, Alicia Cohen, Cindy W Leung

**Affiliations:** 1 Department of International Health, Johns Hopkins University Bloomberg School of Public Health, Baltimore, MD 21205, USA; 2 Department of Health Management and Policy, University of Michigan School of Public Health, Ann Arbor, MI, USA; 3 Institute for Social Research, University of Michigan, Ann Arbor, MI, USA; 4 Providence VA Medical Center, Providence, RI, USA; 5 Departments of Family Medicine and Health Services, Policy and Practice, Brown University, Providence, RI, USA; 6 Department of Nutritional Sciences, University of Michigan School of Public Health, Ann Arbor, MI, USA

**Keywords:** Food insecurity, First-generation student status, College, Graduation, Educational attainment, Panel Study of Income Dynamics

## Abstract

**Objective:**

To examine the effect of food insecurity during college on graduation and degree attainment.

**Design:**

Secondary analysis of longitudinal panel data. We measured food insecurity concurrent with college enrolment using the 18-question United States Department of Agriculture Household Food Security Survey Module. Educational attainment was measured in 2015–2017 via two questions about college completion and highest degree attained. Logistic and multinomial logit models adjusted for socio-demographic characteristics were estimated.

**Setting:**

USA

**Participants:**

A nationally representative, balanced panel of 1574 college students in the USA in 1999–2003 with follow-up through 2015–2017 from the Panel Study of Income Dynamics.

**Results:**

In 1999–2003, 14·5 % of college students were food-insecure and were more likely to be older, non-White and first-generation students. In adjusted models, food insecurity was associated with lower odds of college graduation (OR 0·57, 95 % CI: 0·37, 0·88, *P* = 0·01) and lower likelihood of obtaining a bachelor’s degree (relative risk ratio (RRR) 0·57 95 % CI: 0·35, 0·92, *P* = 0·02) or graduate/professional degree (RRR 0·39, 95 % CI: 0·17, 0·86, *P* = 0·022). These associations were more pronounced among first-generation students. And 47·2 % of first-generation students who experienced food insecurity graduated from college; food-insecure first-generation students were less likely to graduate compared to first-generation students who were food-secure (47·2 % *v*. 59·3 %, *P* = 0·020) and non-first-generation students who were food-insecure (47·2 % *v*. 65·2 %, *P* = 0·037).

**Conclusions:**

Food insecurity during college is a barrier to graduation and higher-degree attainment, particularly for first-generation students. Existing policies and programmes that help mitigate food insecurity should be expanded and more accessible to the college student population.

Education is a core social determinant of health, and higher educational attainment, particularly a college degree, is associated with numerous health and social advantages across the life course^(1–5)^. In the USA, the high cost of a college education^(6)^ is one of the barriers to students from low-income families being able to successfully enrol in higher education^(4,6–8)^. Due to numerous policy changes in recent decades, low-income students have had greater opportunities to enrol in higher education at both community colleges and 4-year institutions^(4)^. Once enrolled, however, other barriers can prevent students from successfully completing their degree^(4)^. Food insecurity, or the lack of consistent access to enough food for active healthy life^(9)^, is one such barrier that could impact graduation and the type of college degree attained.

Food insecurity is a serious problem among college students in the USA^(10,11)^. Numerous studies conducted at individual or regional groups of institutions have yielded prevalence estimates of food insecurity among college students that vary widely with many estimates over 50 % at some institutions^(10,12–19)^. Food insecurity among college students is consistently higher than food insecurity prevalence in the general population, which, in 2019, was 11 %^(9)^. Though no single study has assessed college food insecurity in a nationally representative sample, best estimates prior to the COVID-19 pandemic are that approximately 33–41 % of college students were food-insecure^(20,21)^. Food insecurity among college students is associated with numerous adverse health and social outcomes, including worse diet quality, mental health, and physical health, and lower grade point average (GPA)/academic performance^(10,12,13,15,16,18,19,22–24)^. Though there is a growing body of research about the determinants of and effects associated with food insecurity, to date, research about college food insecurity has largely been cross-sectional and long-term outcomes have not been assessed.

To our knowledge, this is the first nationally representative, longitudinal study of the effect of food insecurity among college students on educational attainment outcomes. The goal of this study was to examine, using a nationally representative, longitudinal panel survey, the effect of experiencing food insecurity during college, on college completion and type of degree attained. Because first-generation students face many barriers to college graduation and often have worse outcomes than non-first-generation students^(25,26)^, we also examined potential effect modification based on first-generation student status. We hypothesised that students who experience food insecurity while in college would be less likely to graduate and would be less likely to obtain graduate degrees over the 18-year follow-up period.

## Methods

Data were obtained from the Panel Study of Income Dynamics (PSID)^(27)^. The PSID is the world’s longest running nationally representative household panel survey. Since data collection began in 1968, the PSID has followed the original 5000 family sample as well as their descendants^(28)^. Families have also been added to the PSID over time to reflect changes in the composition of the national population. Data collection on socio-demographic, economic and health characteristics were collected annually from 1968 to 1997 and biennially thereafter. In the 1999 wave of data collection, the PSID measured food insecurity status for the first time using the US Department of Agriculture 18-question Household Food Security Survey Module^(29)^. Food insecurity was also measured in the 2001, 2003, 2015 and 2017 data collection waves. Therefore, for this study, we included individuals who were in college in 1999–2003 and followed them through 2017. To construct the analytic sample, we created a balanced panel of 1574 individuals who were enrolled in higher education in at least one data collection wave from 1999 to 2003 and were still in the PSID sample as of 2015 or 2017.

PSID sample members who were attending college were identified using several prospective and retrospective questions including student status as of interview, month and year of high school graduation or GED completion, number of years attended college, dates of college attendance, and whether an individual was a student in the last calendar year. Individuals were also assumed to be attending college if they indicated they had graduated from high school or attained a GED, were currently attending school and were ≥ 20 years old. College students in our sample could be an independent head or spouse/partner of their own household (*n* 306), a dependent family member living at home but attending college (*n* 930), or a dependent family member enrolled in college and living away from home (i.e. in an apartment, or dormitory at school) (*n* 338).

### Measures

Food insecurity was measured using the United States Department of Agriculture 18-question US Household Food Security Survey Module^(30)^. The eighteen questions (or ten questions in households without children) are asked in stages of the primary survey respondent about the household as a whole. Food security status is categorised into four categories: high or full food security, marginal, low, and very low food security. High or full food security, meaning all household members had sufficient access to food at all times, is defined as 0 affirmative responses. Marginal food security, or concerns or worries about insufficient resources for food, is defined as 1–2 affirmative responses. Low food security, or reduction in the quality and variety of food intake, is defined as 3–7 for households with children and 3–5 for households without children. Very low food security, or the reduction of the quantity as well as quality of food consumed, is defined as a score of 8–18 for households with children and 6–10 for households with children. College students in our sample were considered food-insecure if they met the criteria for marginal, low or very low food security status at any time they were in college from 1999 to 2003. Following prior studies, students with marginal food security (*n* 163) were included in the food-insecure group along with those with low and very low food security (*n* 154)^(31,32)^.

Because food insecurity is a household measure, we prioritised direct measurement of the college student’s food insecurity status. Therefore, if at any point from 1999 to 2003, an individual was a head or spouse/partner of their own household while in college, we used that food security score. If an individual was not the head or spouse/partner of their own household from 1999 to 2003, but was living in the home of the primary respondent while in college, we used that food insecurity measure. The remaining individuals were dependents of the primary respondent but lived away at school at the time of interview when they were enrolled in college, so the food security measure is that of the primary respondent’s (i.e. parent/caregiver) household. Following this hierarchy, we coded food insecurity status for each wave in which the individual was a college student. We then created a binary measure of food insecurity from 1999 to 2003 coded as 1 if the individual was ever food-insecure while in college and 0 if they were never food-insecure while in college.

Educational attainment was the outcome measure. We assessed educational attainment based on two household interview questions in the 2015 and 2017 survey waves about whether the respondent completed college and, if so, the highest degree they obtained. We then created a categorical measure of educational attainment with the following four categories: (1) a binary measure of whether or not the individual completed college and (2) a categorical measure of the type of degree they received (no degree, associate’s degree, bachelor’s degree or graduate/professional degree).

Study covariates included mean age while in college, poverty level (income-to-needs ratio) while in college, sex (male and female), race/ethnicity (non-Hispanic White and non-White), first-generation student status (i.e. neither parent had graduated from college) and household position while in college (head or spouse/partner of own household, family member living in the home of the primary respondents (known as ‘Other Family Unit Member’ (OFUM)) and a family member of the primary respondent living away at school (known as ‘Institutional OFUM’).

### Analyses

Complex survey weights accounting for the longitudinal nature of the data, sample attrition, clustering, and strata were used for all analyses to generate estimates that are nationally representative of the US population. We used logistic models to estimate the odds of graduating from college and multinomial logistic models to estimate the odds of the different degree types. For both outcomes, two models were estimated: (1) adjusted for age, sex, race/ethnicity and household position; and (2) adjusted age, sex, race/ethnicity, household position, first-generation student status, and poverty level. Finally, we used a multinomial logistic regression model to estimate the odds of the different degree types, including an interaction term between college food insecurity status and first-generation status with further adjustment for the other covariates included in Model 2 above. We used post-estimation margins to generate the predicted probability of being in each level of the outcome based on the interaction between food insecurity and first-generation status while holding all other variables at their means. Significance was considered at *P* < 0·05 and all tests were two-sided. Analyses were conducted in 2020, and the survey software Stata version 15 was used.

## Results

Characteristics of the study sample are shown in Table 1. During the college period of 1999–2003, the mean age was 21·6 (±0·13) years. The majority of adults were female (54·5 %), non-Hispanic White (73·9 %) and a first-generation college student (54·3 %). The overall prevalence of food insecurity during college was 14·9 %. Individuals who experienced food insecurity in college were more likely to be of older age (*P* < 0·001), non-White (*P* < 0·001) and a first-generation college student (*P* < 0·001), compared to adults who were food-secure in college.

**Table 1:** Characteristics of the study sample, PSID (*n* 1574)

	All		Food-secure		Food-insecure		
	*n*	%	*n*	%	*n*	%	*P*-value
Total	1574	100 %	1257	85·1	317	14·9	
1999–2003							
Age							0·656
Mean	21·62		21·61		21·75		
sd	0·13		0·13		0·31		
Income to needs ratio							< 0·001
Mean	5·23		5·78		2·18		
sd	23·3		0·26		0·13		
Sex							
Male	648	45·5	526	45·8	122	43·7	0·64
Female	926	54·5	731	54·2	195	56·3	
Race/ethnicity							
Non-Hispanic White	892	73·9	796	78	96	50·7	< 0·001
Other	562	16·2	368	12·3	194	38·5	
First-generation student							
Yes	959	54·3	699	50·4	260	77	< 0·001
No	615	45·7	558	49·6	57	23	
Household position							
Head/spouse/partner	306	21·6	234	20	72	30·4	< 0·001
Institutional OFUM	338	22·6	297	24·8	41	10·2	
OFUM	930	55·8	726	55·2	204	59·3	
2015/2017							
College degree completion							
Yes	939	64·5	805	68·1	134	43·8	< 0·001
No	635	35·5	452	31·9	183	56·2	
Highest degree achieved							
Associate’s degree	189	11·4	151	10·9	38	13·9	< 0·001
Bachelor’s degree	472	33·6	411	35·8	61	21·1	
Graduate/professional degree	263	19·2	233	21·2	30	7·6	

OFUM, Other Family Unit Member.

*P*-values are from weighted chi-squared tests.

In bivariate analyses, college food insecurity was inversely associated with college degree completion and educational attainment. Among food-insecure college students, 43·8 % completed their college degree compared with 68·1 % of food-secure college students (*P* < 0·001). Among college students who completed a degree, those who experienced food insecurity were more likely to get an associate’s degree (13·9 % *v*. 10·9 %) and were less likely to receive a bachelor’s (21·1 % *v*. 35·8 %) or graduate/professional degrees (7·6 % *v*. 21·2 %) than their food-secure counterparts.

Table 2 presents adjusted associations between college food insecurity and college completion and type of degree attained. After adjustment for age, sex, race/ethnicity and household position, food insecurity during college was associated with lower odds of college completion (OR 0·46, 95 % CI 0·30, 0·70, *P* = 0·001), and lower likelihood of obtaining a bachelor’s degree (relative risk ratio (RRR) 0·45, 95 % CI 0·30, 0·69, *P* < 0·001) or a graduate or professional degree (RRR 0·25, 95 % CI: 0·12, 0·55, *P* = 0·001). After further adjustment for first-generation status and poverty level, college food insecurity remained significantly associated with lower odds of college graduation (OR 0·57, 95 % CI: 0·37, 0·88, *P* = 0·013) and lower likelihood of obtaining a bachelor’s degree (RRR 0·57, 95 % CI: 0·35, 0·92, *P* = 0·022) or graduate/professional degree (RRR 0·39, 95 % CI: 0·17, 0·86, *P* = 0·022). Independent of food security status, first-generation status was also a significant predictor of college completion and degree attainment. First-generation students were less likely to graduate from college (OR 0·44, 95 % CI: 0·31, 0·62, *P* < 0·001) and less likely to obtain a bachelor’s degree (RRR 0·43, 95 % CI: 0·29, 0·62, *P* < 0·001) or graduate/professional degree (RRR 0·21, 95 % CI: 0·13, 0·35, *P* < 0·001).

**Table 2:** Associations between food insecurity and college completion and degree attained, PSID (*n* 1574)

	Model 1			Model 2		
Food insecurity	OR	95 % CI	*P*-value	OR	95 % CI	*P*-value
College degree completion						
Yes	0·461	0·304, 0·701	0·001	0·574	0·374, 0·882	0·013
Highest degree achieved*	RRR	95 % CI	*P*-value	RRR	95 % CI	*P*-value
Associate’s degree	0·662	0·308, 1·422	0·281	0·633	0·301, 1·331	0·220
Bachelor’s degree	0·453	0·299, 0·689	< 0·001	0·568	0·353, 0·916	0·022
Graduate/professional degree	0·253	0·116, 0·552	0·001	0·388	0·174, 0·864	0·022
First-generation status						
College degree completion	OR	95 % CI	*P*-value	OR	95 % CI	*P*-value
Yes				0·435	0·307, 0·615	< 0·001
Highest degree achieved*	RRR	95 % CI	*P*-value	RRR	95 % CI	*P*-value
Associate’s degree				1·114	0·689, 1·887	0·601
Bachelor’s degree				0·428	0·294, 0·623	< 0·001
Graduate/professional degree				0·213	0·130, 0·348	< 0·001

RRR, relative risk ratio.

Model 1: Logit (for degree completion) and multinomial logit (for degree type) models adjusted for age, sex, race/ethnicity and household position (HSP, Institutional OFUM).

Model 2: Logit (for degree completion) and multinomial logit (for degree type) models adjusted for Model 1 covariates plus first-generation students status and income to needs ratio.

Food insecurity defined as marginal, low and very low food security status.

* Fifteen people are missing degree information.

**Fig. 1:**
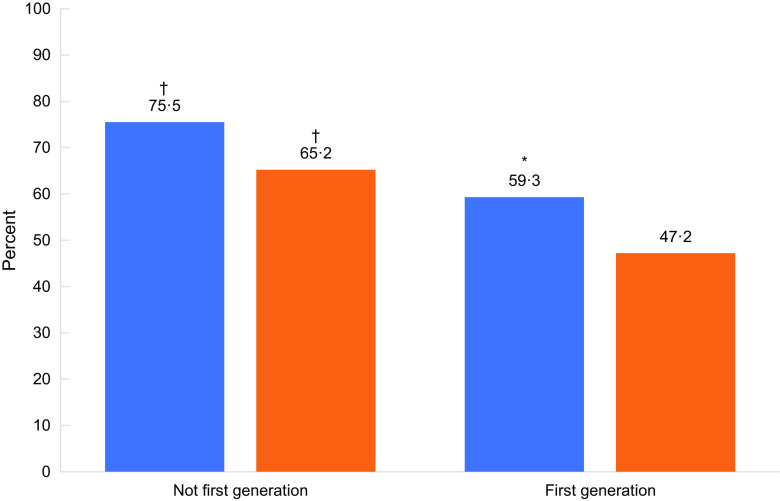
Predicted probability of college degree completion by food security and first-generation student status: 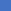, food-secure; 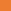, food-insecure. Note: Post-estimation margins from the interaction between food security and first-generation status from a logit model including an interaction between food security status and first-generation status adjusted for household position, age, sex, race and income to needs ratio. *Differences between food secure and food insecure (within first-generation status) significant at *P* < 0·05. †Differences between first-generation status (within food security status) significant at *P* < 0·05

The predicted probability of college degree completion based on adjusted models with an interaction between food security and first-generation status is shown in Fig. 1. Less than half of first-generation students who experienced food insecurity during college graduated from college (47·2 %). Among students who were first-generation, food-insecure students had significantly lower odds of college completion (47·2 % *v*. 59·3 %, *P* = 0·02). First-generation students experiencing food insecurity were also more likely than non-first-generation students experiencing food insecurity to not complete college (47·2 % *v*. 65·2 %, *P* = 0·037).

Among food-secure students, first-generation status was associated with lower probability of graduating from college (59·3 % *v*. 75·5 %, *P* < 0·001). Among students who were not first generation, food security status was not significantly associated with differences in college completion.

**Fig. 2:**
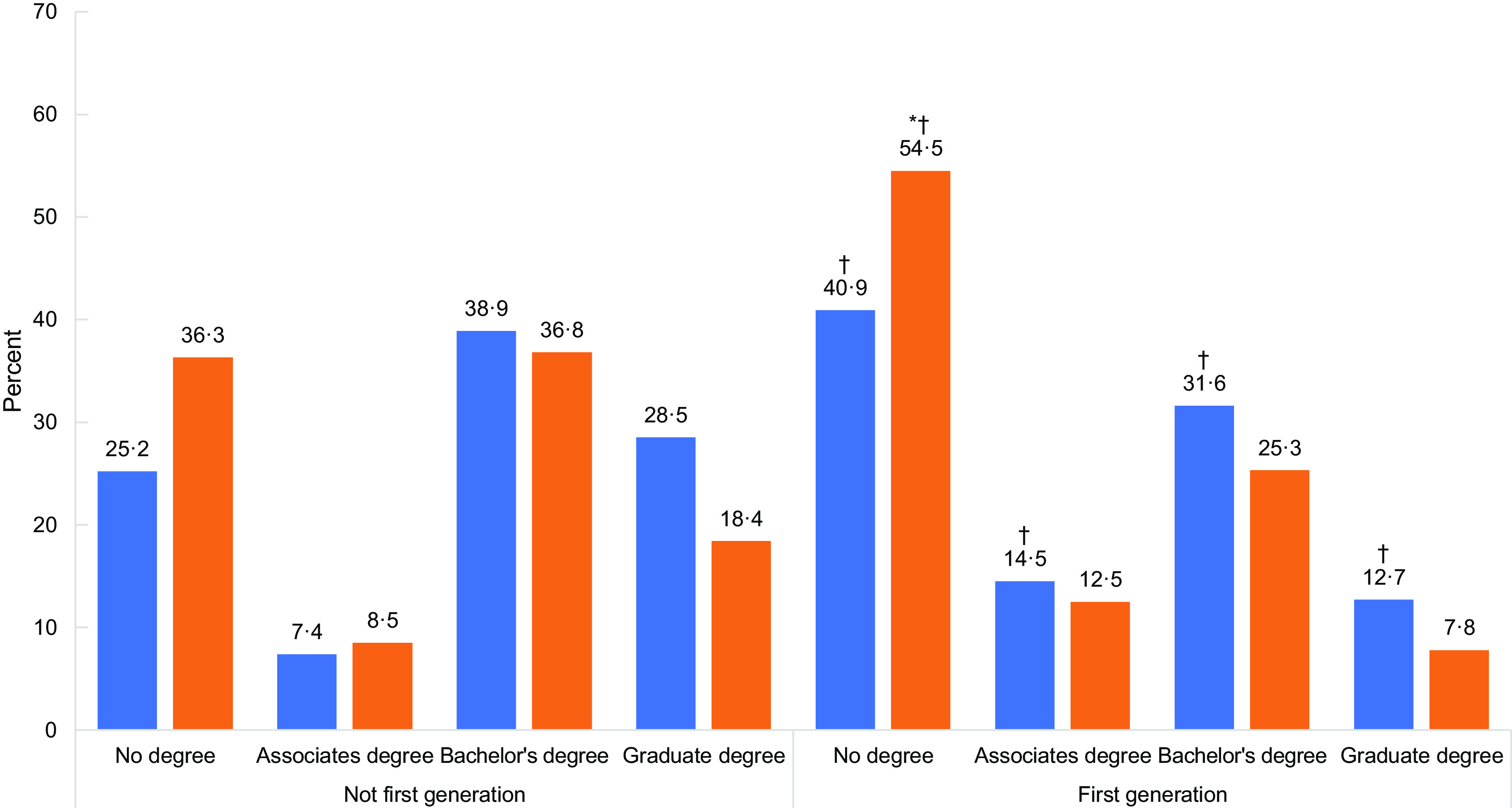
Predicted probability of type of degree completed by food security and first-generation status: 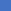, food-secure; 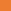, food-insecure. Note: Results are post-estimation margins after a multinomial logit model including an interaction between food security and first-generation status and adjusted for household position, age, sex, race and income to needs ratio. *Within each degree outcome, differences between food-secure and food-insecure (within first-generation status) significant at *P* < 0·05. †Within each degree outcome, differences between first-generation status (within food security status) significant at *P* < 0·05

Figure 2 displays predicted probabilities of college degree outcomes from the adjusted multinomial logistic models containing an interaction between food insecurity status and first-generation status. Food-insecure college students who were first generation were the least likely to graduate at all (54·5 %) and were significantly less likely than their food-secure, first-generation counterparts to graduate (54·5 % *v*. 40·9 %, *P* < 0·001). Food-insecure college students who were first generation were also the least likely to receive a bachelor’s degree (25·3 %) or graduate/professional degree (7·8 %), though differences based on food security status were not statistically significant.

## Discussion

In this study we describe results from a large, nationally representative, longitudinal panel survey regarding the effect of food insecurity during college on college completion and type of degree attained. To our knowledge, this is the first study to examine the effect of food insecurity during college on graduation and degree attainment in a nationally representative sample using prospective data. We find that experiencing food insecurity during college is associated with lower odds of college completion, particularly for food-insecure students who are also first-generation college students. We also find that food-insecure students who do graduate are more likely to receive an associate’s degree and are less likely to receive a bachelor’s degree or graduate/professional degree than their food-secure counterparts. Given the importance of a college degree for economic mobility^(4)^, and the effect of different degree types on future employment and income, these findings underscore an important facet of the lasting negative impact of experiencing food insecurity during college, particularly for first-generation students who already face substantial obstacles to graduating from college^(25,26)^.

Historically, for those who can access higher education, college has been a key pathway out of poverty via access to higher paying jobs and more stable employment for college graduates^(4,8,33)^. The importance of a college degree for upward social mobility is particularly marked for low-income and first-generation college students^(4,7)^. Attainment of a college degree has traditionally led to higher earning potential, better economic stability, higher socio-economic status, and numerous other positive outcomes including better health^(5,19,33,34)^. However, many students, particularly those who are low-income and first-generation college attendees, face barriers to graduating from college once they enrol which put them at risk of taking on the expense and debt of a college education without being able to then reap the benefits of obtaining a degree^(4,8)^. The present findings underscore how the experience of food insecurity during college can be a substantial barrier to graduation and attainment of bachelor’s and graduate/professional degrees; thus contributing to societal inequalities and health disparities linked to education by impeding the ability of food-insecure students to attain the upward social mobility a college degree confers.

There are several avenues through which the experience of food insecurity during college could contribute to lower graduation rates and lower odds of bachelor’s and graduate/professional degrees. First, worrying about not having enough to eat, where your next meal is coming from, going hungry, or sacrificing the nutritional content of food can distract students from focusing on school work thereby leading to lower academic performance^(18,19,22,23)^. A recent study of first-year college students found that students experiencing food insecurity had lower GPA even adjusting for high school academic performance and socio-economic status^(24)^. Other literature among college students has shown a strong association between food insecurity and lower academic performance including lower GPA, difficulty concentrating during class, and higher likelihood of withdrawing from classes or not returning to school the following year^(10,16,23,24,35–37)^. Second, food insecurity can isolate students from their peers and contribute to feelings of stigma, shame, not belonging or not being supported^(38)^. Feelings of not belonging or being out of place within the college student community are already common among students who are most at risk for food insecurity including first-generation students and underrepresented racial/ethnic minorities on some college campuses^(8)^. Third, students who experience food insecurity are more likely to be working to help support themselves (i.e. pay rent and pay for other living expenses), which limits the amount of time they can devote to their studies. Food-insecure students may also need to work longer hours which can add to the stress of balancing work and school responsibilities. The fact that bachelor’s degrees are a prerequisite for graduate and professional degrees is an additional barrier to low-income students at risk for food insecurity accessing higher degrees associated with high-paying careers.

In the present study, the prevalence of food insecurity observed among college students in 1999–2003 was 14·9 %. This is substantially lower than estimates of food insecurity among more recent samples of college students which vary widely but are often over 50 %^(10,11)^. In a recent review by Nikolaus et al., the authors estimate a prevalence of food insecurity among college students, weighted across all studies, of 41 %^(21)^. In the past several decades, access to a college education has expanded for low-income students even as the cost of college has risen^(8,11)^. For example, from 1999–2000 to 2016, the percentage of college of students who were low-income approximately doubled from 23 % to about 40 %^(11)^. In addition to a higher proportion of low-income students at US colleges and universities, the higher cost of college may contribute to the high prevalence of food insecurity among students in recent years. The cost of a college education has risen substantially at community colleges as well as public and private 4-year institutions over time, and financial support for students and their families has not kept pace^(6,11)^.

The Supplemental Nutrition Assistance Program (SNAP), formerly known as food stamps, is the largest federal nutrition assistance programme in the USA and provides money to low-income Americans to purchase food. SNAP has been shown to improve food security among participants^(39)^. However, SNAP also has numerous requirements for participation that limit accessibility for college students. In particular, SNAP places strict work requirements on able-bodied adults without dependents (ABAWD) that prevent many full-time students from being able to access SNAP benefits. Students who are eligible to receive SNAP benefits often do not receive them as SNAP eligibility rules and applications are complicated, and many students may not realise they are eligible to receive benefits^(40)^. A recent analysis showed that, in 2016, almost 2 million students who were potentially eligible to receive SNAP benefits did not receive them^(11)^. Provisions in the recent spending bill and COVID-19 relief package passed at the end of December 2020 expanded SNAP eligibility for college students by waiving work requirements for students eligible for federal or state work-study programmes^(41)^. These changes will make nearly 3 million college students newly eligible to receive SNAP benefits^(42)^. Many students many not be aware that they are newly eligible, so outreach and clear communication about current SNAP eligibility rules and how to apply is imperative. However, these changes are only temporary and are currently set to expire in September 2021, so longer-term solutions for helping low-income college students access SNAP are needed.

College students’ lives have been highly disrupted by COVID-19, and it is likely that food insecurity in this population has only grown over the course of the pandemic^(17)^. Many college campuses have moved to virtual instruction, campus housing is limited or unavailable, and industries (i.e. the service and hospitality sectors) that provide employment to college students have been particularly hard-hit limiting employment opportunities for the college student population. Food insecurity has risen to unprecedented levels during the pandemic, and young people and people without a college degree have been particularly hard-hit^(43)^. Longer-term policy changes at multiple levels, federal, state and college/university are needed to address food insecurity among college students^(40,44,45)^. Policy changes that ensure that college students can continue to access needed SNAP benefits for the duration of the economic effects of the pandemic and beyond should be a particular priority. State and federal policies to address food insecurity should also carefully consider the accessibility of food assistance programmes to college students. Actions by colleges and universities to support students and connect them with needed resources can also help promote food security for all students. Schools can facilitate connecting students with local community resources, can help provide transportation options to grocery stores and can also create on-campus resources such as food pantries.

However, increasing access to SNAP, food pantries and other community food resources will not address the underlying causes of food insecurity among college students and the impact on improving college graduation rates and degree attainment for food-insecure students may be limited. State and federal policy-makers should consider incentivising school-level investments in robust programmes that support low-income students to help them to succeed academically and meet their basic needs, including access to sufficient healthy food, while in school^(40)^. Relatedly, greater investment in college readiness programmes at the high school level particularly for low-income and first-generation students is also warranted^(7)^. Additionally, investment in policies and programmes that address the rising cost of college and help low-income and first-generation college students to afford the full cost of college (i.e. tuition and living expenses) are urgently needed, so low-income students can get a college education without taking on huge debts.

This study has several strengths. This is the first nationally representative, longitudinal study of the effects of food insecurity during college on educational outcomes. Prior studies have been cross-sectional and have only been able to examine contemporaneous associations between food insecurity and academic performance. Prior studies have also generally taken place in the samples of students at a single institution or in several institutions in a particular region of the country. In the present study, we use a large, nationally representative longitudinal panel, with detailed financial information for the students and their families that includes the gold standard measure of food insecurity (18-question United States Department of Agriculture Household Food Security Survey Module) and a long follow-up period (18 years). In addition, our sample includes students at community colleges as well as public and private 4-year institutions, which has been a limitation of prior research.

In addition to these strengths, our results should also be considered in light of several limitations. First, for college students who were living away from their parents, but who were not yet financially independent from their parents (*n* 338), the measure of food insecurity in the PSID reflected that of the parent’s household, not the household in which the student was living when they were away at school. This indirect measure of food insecurity may not be an accurate representation of the student’s food security status while in college. However, for the majority of our sample, we do have a direct measure of their food security status for the household in which they are living whether they are the head of their own household and independent from their parents, or if they were living at home in their parent’s household while attending college. Second, we used a binary measure of food insecurity that did not allow us to examine differential effects based on severity of food insecurity experienced during college. While such an approach would be informative and is an important direction for future research, sample size limitations and the complexity of capturing food security status across three waves of data collection and multiple household positions prevented us from taking this approach in the present study. Third, our sample of college students attended college from 1999 to 2003 and may not be representative of the current or more recent college student population. 1999, 2001 and 2003 were the first data collection waves in which the PSID collected food insecurity information, and food insecurity was not measured again until 2015. Therefore, in order to examine the longitudinal effect of food insecurity during college on degree attainment, we used the earlier cohort of college students with food insecurity measures. Given substantial increases in college tuition, and expansion of college access to lower-income students in recent years, this may partially explain why our overall prevalence of food insecurity among college students (14·5 %) is lower than other, more current estimates^(10,14,20)^. In our sample, as in other more recent samples, food-insecure students were more likely to be low income, non-White and first-generation^(10,16,21)^. It is worth noting that there has been wide variation in how food security status is measured among college students and dispute about how well food security measures intended for the general population capture food insecurity among college students^(46)^. The measure used in this study has performed well among college students in comparison to other measures of food insecurity, but more research about how to best capture food insecurity among college students is needed^(46)^.

## Conclusion

This is the first longitudinal, nationally representative study of the effect of college food insecurity on college graduation and degree attainment. Students experiencing food insecurity, particularly those who are first-generation students, are less likely to graduate from college, and if they do graduate, they are more likely to receive an associate’s degree rather than a bachelor’s or graduate/professional degree after 18 years of follow-up. Given the importance of education and educational attainment as social determinants of health, these findings underscore an important pathway through which the experience of food insecurity during college can have long-term adverse effects throughout the life course. Policies and programmes at the federal, state and college/university levels are urgently needed to address food insecurity during college.
